# Rising Background Odor Concentration Reduces Sensitivity of ON and OFF Olfactory Receptor Neurons for Changes in Concentration

**DOI:** 10.3389/fphys.2016.00063

**Published:** 2016-03-01

**Authors:** Maria Hellwig, Harald Tichy

**Affiliations:** Department of Neurobiology, Faculty of Life Sciences, University of ViennaVienna, Austria

**Keywords:** odor concentration coding, ON and OFF responses, asymmetric sensitivities, effect of background concentration, gain of responses

## Abstract

The ON and OFF ORNs on cockroach antennae optimize the detection and transfer of information about concentration increments and decrements by providing excitatory responses for both. It follows that the antagonism of the responses facilitates instantaneous evaluations of the odor plume to help the insect make tracking decisions by signaling “higher concentration than background” and “lower concentration than background”. Here we analyzed the effect of the background concentration level of the odor of lemon oil on the responses of the ON and OFF ORNs to jumps and drops of that odor, respectively. Raising the background level decreases both the ON-ORN's response to concentration jumps and the OFF-ORN's response to concentration drops. Impulse frequency of the ON ORN is high when the concentration jump is large, but for a given jump, frequency tends to be higher when the background level is low. Conversely, impulse frequency of the OFF cell is high at large concentration drops, but higher still when the background level is low. Analyses of this double dependence revealed that the activity of both types of ORNs is raised more by increasing the change in concentration than by decreasing the background concentration by the same amount. This effect is greater in the OFF ORN than in the ON ORN, indicating a bias for falling concentrations. Given equal change in concentration, concentration drops evoke stronger responses in the OFF ORN than concentrations jumps in the ON ORN. This suggests that the OFF responses are used as alert information for accurately tracking.

## Introduction

An insect tracking a turbulent odor plume to its source perceives the odor signal as a sequence of pulses of high concentration interspersed with the surrounding medium containing gaps of low or zero concentration (Moore and Atema, 1991; Zimmer-Faust et al., 1995; Vickers, 2000). Key features pertaining to the location of an odor source are the timing of concentration jumps and concentration drops as well as the level of odor concentration between these changes, referred to here as background concentration. The effect of the background value on the responses of ORNs to superimposed concentration pulses was first investigated in the lobster (Borroni and Atema, 1988) and more recently described in the housefly (Kelling et al., 2002). Those experiments tested single synthetic compounds, and the analysis was limited to the concentration jump at the onset of the pulse. While with increasing background level the response of lobster ORNs to concentration jumps gradually diminished, the response of housefly ORNs was enhanced to small jumps and reduced to large jumps. The response enhancement was interpreted as the result of depolarization or diminution of the resting membrane potential due to the presence of the background odor. The response reduction was attributed to competition between stimulus and background molecules for membrane receptors (Borroni and Atema, 1988; Kelling et al., 2002).

Based on these studies, two statements can be made. The first is that ORNs are detectors for the relative rather than the absolute concentration. The second is that the higher the background level, the weaker the responses to rapidly increasing odor concentration. An animal tracking a turbulent odor plume will therefore perceive the same concentration jump progressively weaker the closer it approaches the source. However, weak responses signify less sensory evidence than strong responses. Such constraint has been described in the neural circuits implementing the binary decision about the direction of a motion stimulus, which determines saccadic eye movement in the rhesus monkey (Roitman and Shadlen, 2002; Bogacz et al., 2009). The speed of decision is associated with integrator neurons in pre-motor brain areas which gradually increase their discharge rate by accumulating the inputs of sensory neurons over time. With decreasing difference between the baseline activity of these integrator neurons and the response threshold of non-integrator neurons, decisions are prone to errors. Simply sampling of information sequentially facilitates accurate detection and decision-making under uncertain conditions (Heitz and Schall, 2012; Heitz, 2014). However, sampling as little as possible will save time and effort (Drugowitsch and Pouget, 2012). In view of the situation of the olfactory system, it would be a possible economical alternative to create a separate system of ORNs with a different coding mechanism. Such a strategy of sensory coding and information processing seems to be realized in the ON and OFF ORNs on the cockroach's antennae: they produce opposite responses to changes in odor concentration (Hinterwirth et al., 2004; Tichy et al., 2005; Burgstaller and Tichy, 2011, 2012). The discharge rate of the ON ORNs is increased by raising odor concentration and decreased by lowering it, and the discharge rate of the OFF ORNs is increased by lowering odor concentration and decreased by raising it. During moment-to-moment contact with the odor signal, the activity of the ON ORN increases when the odor concentration jumps to a higher value when contact is made with an odor pulse. Conversely, the activity of the OFF ORN increases when odor concentration drops to a lower value after encountering an odor gap. A recent study reveals a bias for concentration drops, suggesting a bias for detecting the loss of contact with the odor signal (Burgstaller and Tichy, 2011).

In this study, we quantified the simultaneous dependence of the ON and OFF ORNs on the background level and the superimposed concentration jumps or drops of the same odor, respectively. We used the complex odor of lemon oil emanating from citrus fruits as odor stimulus and we determined the gain of response for the background concentration and for the jumps or drops in concentration. In particular we asked: (i) what is the difference between the two gain values in each type of ORN, and (ii) what is the difference between the two types of ORNs? A falling-concentration bias results in overestimation of concentration drops relative to jumps. In terms of accuracy, a drop would be perceived by the cockroach as being larger than it actually is. We examined whether the disparity between the ON and OFF responses to equal concentration jumps and drops depends on the amplitude of the change and the background level. A disparity for larger concentration changes at higher background levels would be an advantage for receiving information about large jumps at the lateral edges of the plume than small jumps within the odor plume. Asymmetry in the neural coding of concentration jumps and drops is at the root of understanding what characteristics of the odor signal are important for tracking a turbulent odor plume.

## Materials and methods

An adult male cockroach was anesthetized with CO_2_ and fixed on a Perspex holder with strips of Parafilm wrapped around the holder. The antenna was fastened with adhesive tape and dental cement on a Perspex stage projecting from the holder. Action potentials were recorded extracellularly with electrolytically sharpened tungsten electrodes. One electrode was placed lengthwise into the tip of the antenna and the other was inserted into the base of the sensillum. The recorded signals were amplified (NPI, SEC-05X) and filtered (0.1–3 kHz), passed through a CED 1401plus (Cambridge Electronic Design, Cambridge, UK; 12 bit, 10 kHz) interface connected to a PC for on-line recording. Spikes were detected and classified off-line using commercial software (spike2, version 6). Impulse frequency (imp/s) is the per-second impulse count for fixed periods of 0.2 s.

The gain of response is defined as the ratio of output to input and given by the slopes of the regression planes that approximate the relation between impulse frequency, background concentration and concentration change (*F* = *y*_*0*_ + *aC* + *b*Δ*C*; where *F* is the impulse frequency and *y*_*0*_ the height of the regression plane, *a* is the background concentration and *b* the concentration change). The *R*^2^ coefficient of determination indicates how well the regression plane approximates the real data points.

The odor of lemon oil (Art. 5213.1; Carl Roth GmbH + Co.KG; Karlsruhe, D) was applied by an air stream merging at 2 m/s from a glass tube 7 mm in diameter. An air dilution olfactometer was used to control odor concentration (Burgstaller and Tichy, 2011). Compressed clean air was divided into two streams and their flow rates were controlled by passing them through mass flow meters. Each stream was led through a 25-l tank; the first tank contained the liquid odorant and the second tank was empty. After flowing out from the tank, each air stream was passed through an electrical proportional valve (Kolvenbach KG, KWS ¾) and an air flow sensor (AMW 3000; Honeywell). The two streams were then combined. In order to hold the total flow rate of the combined air stream constant, the phase of the control voltages of the proportional vales was shifted by 180°. Instantaneous odor concentration was determined by the flow rate ratio of the odor-saturated air to clean air and indicated by the percentage of saturated air in the air steam playing on the antenna. The amplitude of the concentration change was described by the difference between the background level and concentration of the odor pulse. A positive value (+Δ*C*) indicates a concentration jump and a negative value (−Δ) a concentration drop. After adaptation for 30 s to a constant background level, there followed a series of concentration changes to various higher or lower concentrations, each of which was maintained for 1 s before the return to the background value. The steps were presented every 30 s. This paradigm enabled testing at least four series of concentration jumps or concentration drops from different background levels on each of 13 ON and OFF ORNs, respectively.

## Results

**Figure 1:**
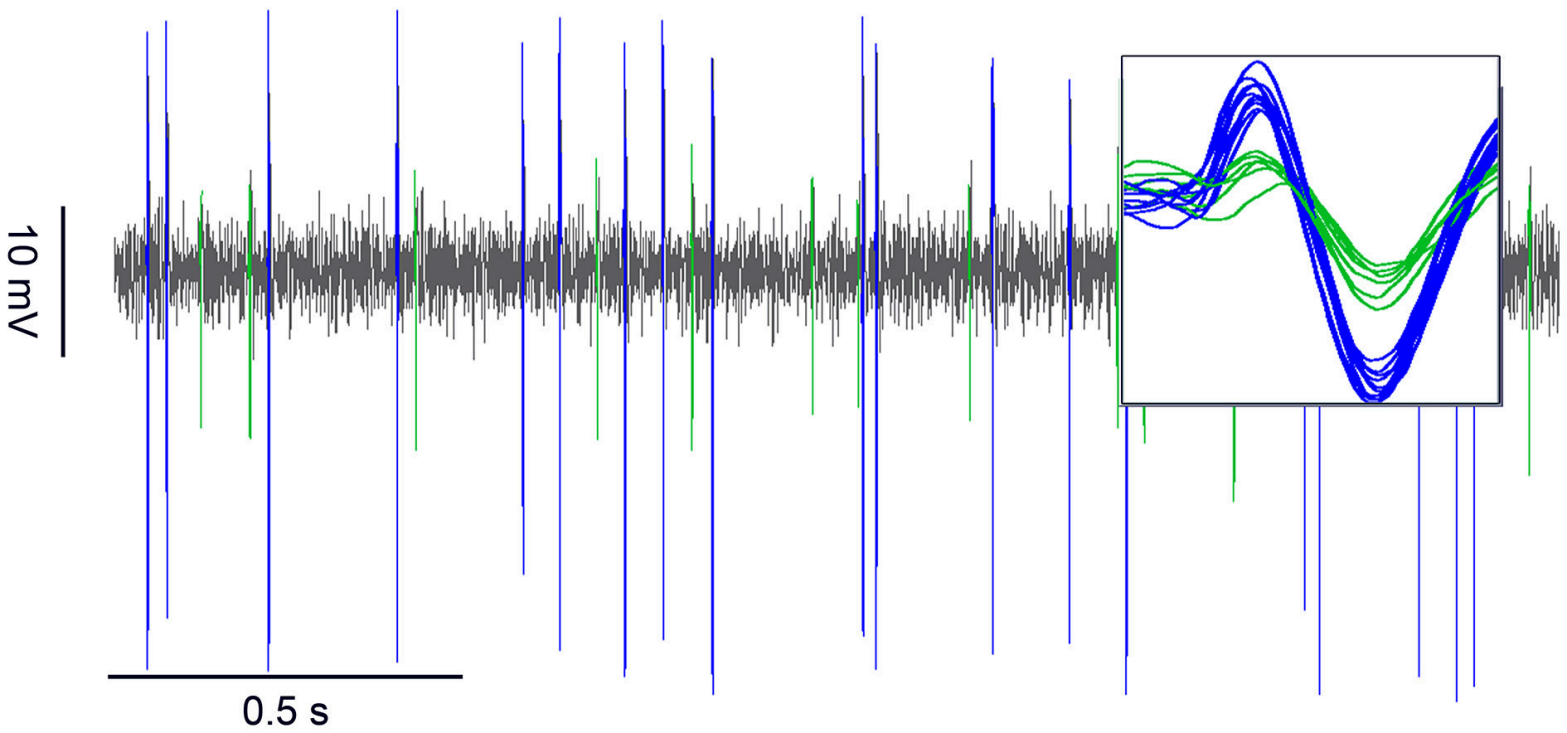
**Simultaneously recorded discharge rates of a pair of ON and OFF ORNs from a single sensillum exposed to the odor of lemon oil at constant concentration of 20%**. The ON ORN (green) displayed smaller impulse amplitudes than the OFF ORN (blue). *Inset* The waveform sensitive template mechanism (spike2) was used to identify and sort action potentials from ON and OFF ORNs.

The ON and OFF ORNs occur together in short, slightly curved hair-like sensilla on the distal margin of each antennal segment. Both ORNs were encountered simultaneously by penetrating the recording electrode gently into the sensillum base. The recordings usually contained the impulses from both ORNs, which could be easily separated by their amplitudes (Figure 1). The impulse trains were sorted into the responses of the ON and OFF ORNs by using a waveform-sensitive template matching mechanism (Figure 1, inset).

**Figure 2:**
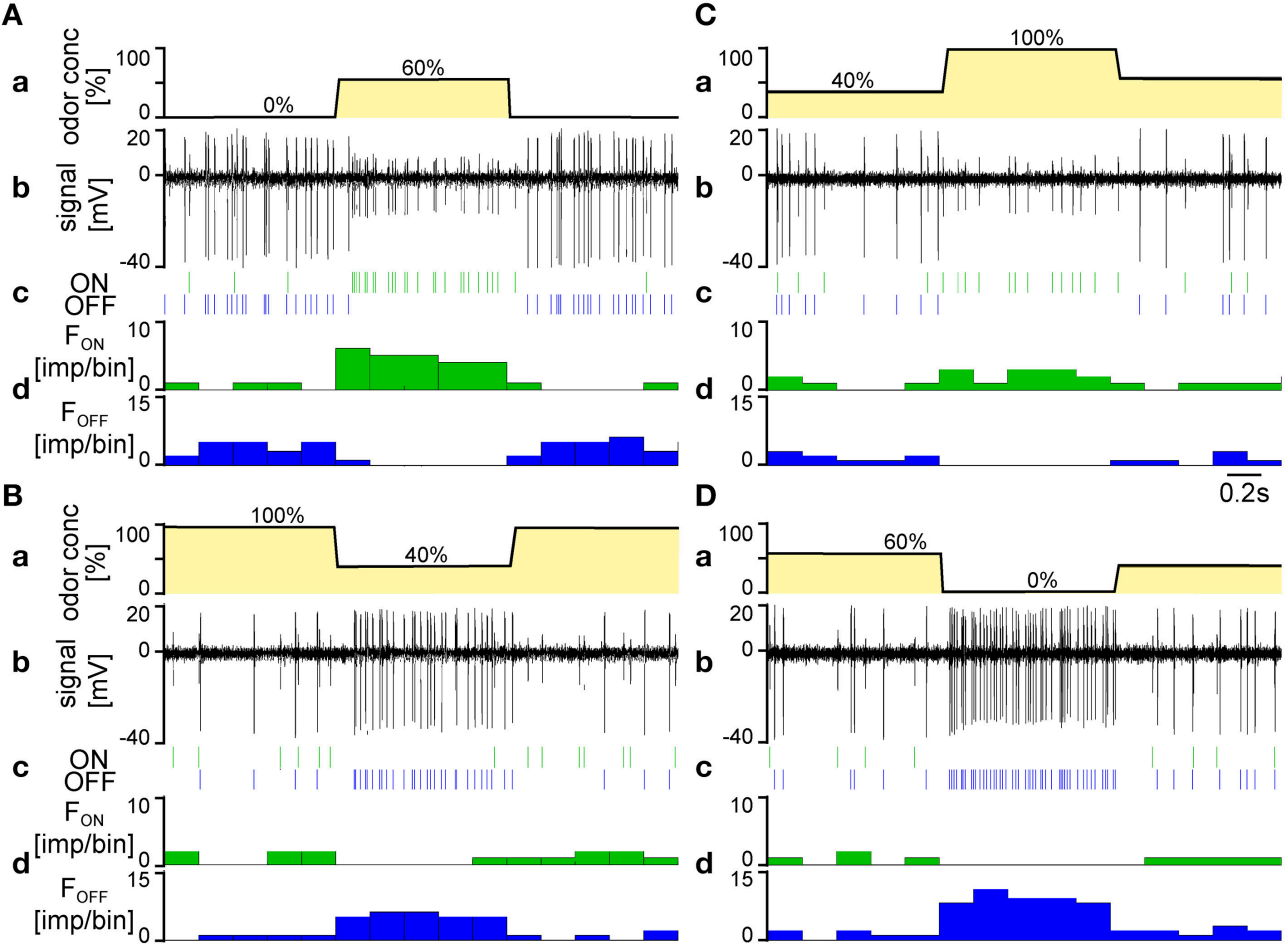
**Simultaneously recorded responses of an ON ORN and an OFF ORN during the final 1-s of a 30-s presentation of different constant background concentration of the lemon oil odor, followed by a 1-s presentation of higher or lower odor concentrations and a return to the initial background value. (A,B)** Concentration jumps (+60Δ%) from 0 to 40% background level, respectively. **(C,D)** Concentration drops (−60Δ%) from 100 to 60% background level, respectively. *a* time course of odor concentration. *b* extracellular recorded action potentials; the OFF ORN displayed larger impulse amplitudes than the ON ORN. *c* action potentials represented as raster plots. *d* responses ORNs represented as time histograms (bin width, 0.2 s).

The experiment shown in Figure 2 illustrates some of the parameters determining the responses of the ON and OFF ORNs. The experiment involved four different background levels of the lemon oil odor (0, 40, 60, and 100%) as well as two 60% jumps and two 60% drops in the concentration of that odor. The ON ORN responded to the concentration jumps with a phasic increase in impulse frequency followed by a decline during the 1-s pulse period. The frequency of the ON ORN was higher at the low (Figure 2A) than at the high background level (Figure 2B). The OFF ORN fell silent for the pulse period. The impulse frequency of the OFF ORN, in contrast, rose rapidly at an odor gap, followed by a decline during the 1-s gap period. Similarly to the ON ORN, the frequency of the OFF ORN was higher at the low (Figure 2D) than at the high level (Figure 2C). The ON ORN ceased discharging during the gap period.

**Figure 3:**
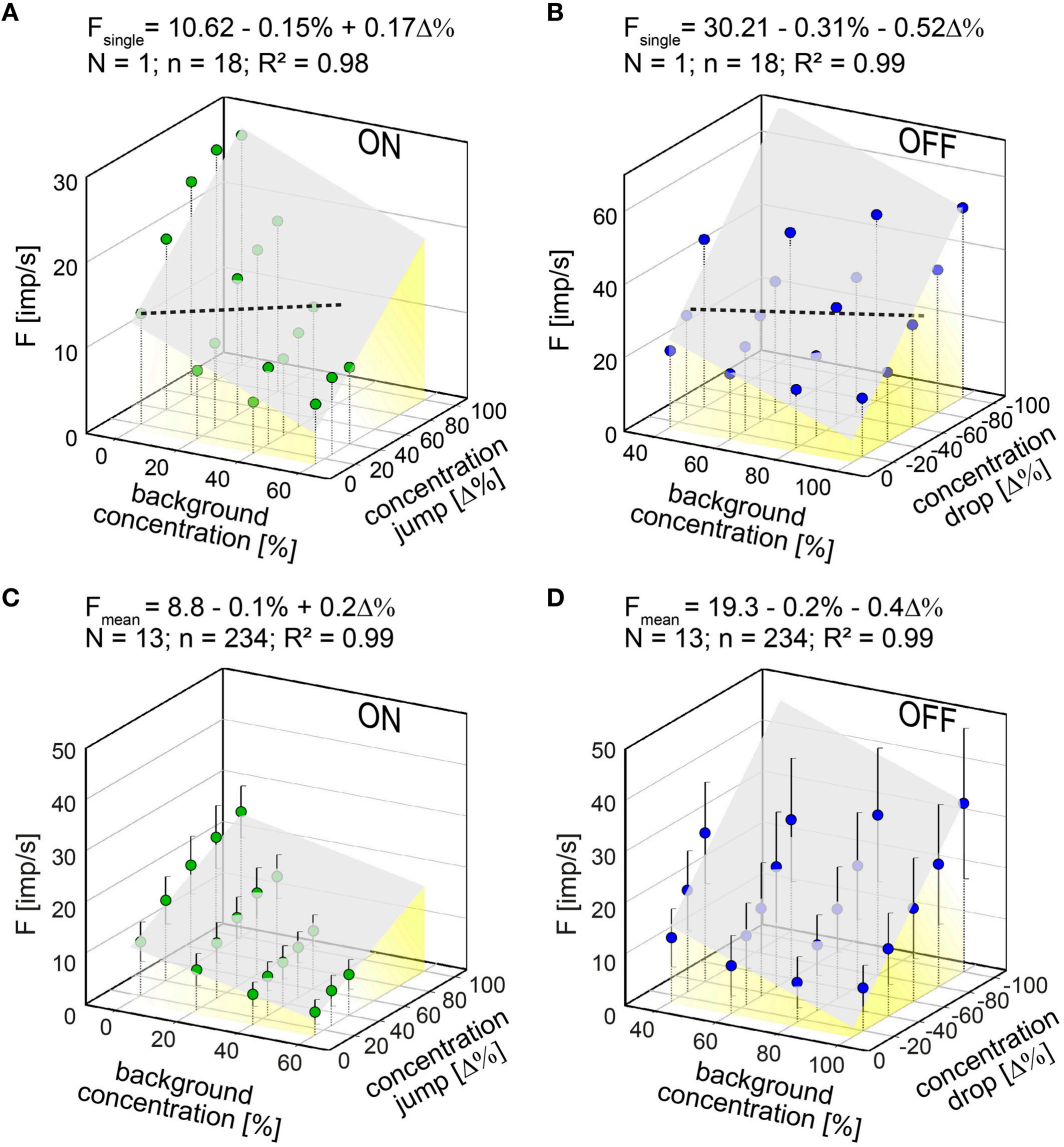
**(A,B)** Responses of a single ON ORN **(A)** and a single OFF ORN **(B)** plotted as a function of background concentration and jumps or drops in odor concentration, respectively. Dotted horizontal lines: equal frequency line at 10 imp/s for the ON ORN and 30 imp/s for the OFF ORN. **(C,D)** Mean responses of 13 ON ORN **(C)** and 13 OFF ORN **(D)** plotted as a function of background concentration and jumps or drops in odor concentration, respectively. Error bars represent SEM. Multiple regressions which utilize 3-dimensional planes (*F* = *y*_*0*_ + *a*C + *b*ΔC; where *F* is the impulse frequency, and *y*_*0*_ the height of the regression plane) were calculated to determine the gain for background concentration (*a* slope) and the concentration change (*b* slope) on the response. Note that the sign of the concentration axis in **(A,C)** is oriented in different direction than in **(B,D)**. *N* number of ORNs, *n* number of points used to calculate regression plane **(A,B)** or mean responses **(C,D)**, *R*^2^ coefficient of determination.

To quantify the effect of the background on the ON-ORN's response to concentration jumps, four concentration series were tested at different levels in the 0–40% range. Frequency increased with the amplitude of the jump, but more rapidly the lower the background. As the equal-frequency line in Figure 3A illustrates, it takes a 60% concentration jump to elicit 10 imp/s at 40% level, but only a 13% jump at 0% level.

The effect of the background on the OFF-ORN's response to concentration drops was described by testing four concentration series at different levels between 40 and 100%. The data obtained resembled those from the ON ORN, inasmuch as frequency of the OFF ORN increased with the amplitude of the drops. The increase in frequency was more rapid the lower the background. This relationship is exemplified in Figure 3B. The equal-frequency line indicates that it takes a 64% concentration drop to elicit 30 imp/s at 100% level, but only a 26% drop at 40% level.

Multiple regressions (*F* = *y*_0_ + *aC* + *b*Δ*C*; where *F* is the impulse frequency and *y*_0_ the height of the regression plane) were calculated to determine the simultaneous effect of the background concentration (*a*–slope) and the jump or drop in concentration (*b*–slope) on the frequency of the ON and OFF ORN, respectively. The slopes demonstrate the three properties that characterize both types of ORNs: (*i*) the sign of the *a*–slope is negative for the ON and OFF ORNs—that is, a decrease in the background raises the frequency of both ORNs to concentration changes; (*ii*) the sign of the *b*–slope is positive for the ON ORN and negative for the OFF ORN—that is, an increase in concentration jumps raises the frequency of the ON ORNs and an increase in concentration drops raises the frequency of the OFF ORNs, and (*iii*) the slopes are steeper for the OFF than for the ON ORN—that is, changes in both the background and in the size of the change have stronger effects on the frequency of the OFF than on that of the ON ORN with due consideration of the sign.

In all 13 examined ON ORNs and 13 OFF ORNs the coefficients of determination of the multiple regressions show a strong linear relationship between impulse frequency, the background level and the concentration change (*R*^2^ > 0.95 in Figures 3C,D). The slopes of the regression planes emphasize the gain of responses for the background concentration (*a*–slope) and the concentration change (*b*–slope). In the ON ORN, the mean gain for jumps was 0.2 imp/s per Δ%, and the mean gain for the background was −0.1 imp/s per %. Frequency can be raised more by increasing the jump by yet another percent than by decreasing the background by 1%. Thus, an increase of 1 imp/s can be elicited either by a 5% increase in the concentration jump or by a 10% decrease in the background.

In the OFF ORN, the mean gain for concentration drops was 0.4 imp/s per −Δ% and the mean gain for the background was −0.2 imp/s per %. Frequency can be raised more by increasing the concentration drop by still another percent than by changing the background by 1%. Thus, an increase of 1 imp/s can be elicited either by a 2.5% increase in the concentration drop or by a 5% decrease in the background. The sensitivity of the OFF ORNs for concentration changes superimposed on the background level is twice as high as that of the ON ORNs.

## Discussion

ON and OFF ORNs responding antagonistically to increments and decrements of the same odor have been described so far only in the cockroach (Hinterwirth et al., 2004; Tichy et al., 2005; Burgstaller and Tichy, 2011, 2012). This may be due to technical reasons. First, the odor stimulus used by the cited authors was provided by means of an air dilution olfactometer. This set-up allowed continuous presentation of odor-loaded air and enabled conditioning the OFF ORN to high concentration levels before dropping to low or zero concentration values. Second, a natural odor was used for stimulation instead of single compounds. We do not know, however, which compounds contained in the odor of lemon oil are responsible for eliciting the antagonistic responses.

In the lobster (Borroni and Atema, 1988) and the housefly (Kelling et al., 2002), increasing the background level reduced the responsiveness of ORNs to concentration jumps (Kelling et al., 2002). A similar effect has been observed for the ON ORN of the cockroach (Burgstaller and Tichy, 2011). Furthermore, the OFF ORNs fit well with this observation because the response to concentration drops decreases with increasing background. However, the responses of the ON and OFF ORNs are not mirror images. The responses of the latter span a larger frequency range than the former, which means that the OFF ORNs respond with higher frequencies to concentration drops than the ON ORN to equivalent jumps (Burgstaller and Tichy, 2011).

In this study we determined the gain of responses of the ON and OFF ORNs for background concentration and superimposed changes of the same odor. In the OFF ORNs, the gain values are twice as high as in the ON ORNs. Thus, falling concentration holds greater salience than rising concentration. Furthermore, with increasing background, the disparity between rising and falling values becomes grater. Notwithstanding this difference, the relationship between the gain values for background concentration and for concentration changes are similar in the ON and OFF ORNs: the value for changes in both types of ORNs is twice as high as the value for the background concentrations.

The stronger gain for changes vs. background reflects the significance of the dynamic aspect of the stimulus. Since the dominance of the gain for concentration change increases with the amplitude of the change and decreases with falling background level, the magnitude of response of an ORN cannot be predicted by simply adding background to change values. This conclusion was drawn from the regression functions in Figures 3C,D. By direct comparisons, an ON ORN will respond to an end-value of 80% attained by a 60% jump from a 20% background with 18.8 imp/s, but to the same end-value of 80% attained by a 20% jump from an 80% background with 4.8 imp/. An OFF ORN will respond to an end-value of 20% attained by an 60% drop from a 80% background with 27.3 imp/, but to the same end-value of 20% attained by a 20% drop from a 40% background with 19.3 imp/s.

Another conclusion from Figures 3C,D is that the background concentration set limits to the dynamic responses of both types of ORN. With increasing background concentration, equal increments in jumps or equal decrements in drops do not cause equal increments in the rate of discharge of the ON and OFF ORNs. Instead, the increments in the discharge rate of both ORNs become progressively smaller. This compressed scaling has some advantages. An ON ORN whose sensitivity to concentration jumps is best at low backgrounds, and decreases as the background from which the jump to be detected increases, provides strong sensory evidence when the cockroach encounters an odor plume. At low backgrounds it will be important to have available a wider dynamic frequency range in order to differentiate between small-amplitude jumps. The same small differences at high backgrounds could be trivial. Conversely, an OFF ORN whose sensitivity to concentration drops is best at low backgrounds, and decreases as the background from which the drop to be detected increases, provides strong sensory evidence at large concentration decreases, when the cockroach approaches the lateral edge of the plume or even leaves the plume. At low backgrounds it will be important to have available a wider dynamic frequency range in order to distinguish between small-amplitude drops. The same small differences at high backgrounds may be less important. Nonetheless, small-amplitude differences may bear a vital message too. Therefore, there must be some mechanism to secure the information conveyed by responses which become progressively weaker and prevent loss of contact with the odor signal. Such a mechanism seems to be realized by the bias of the antagonistically responding ON and OFF ORNs.

**Figure 4:**
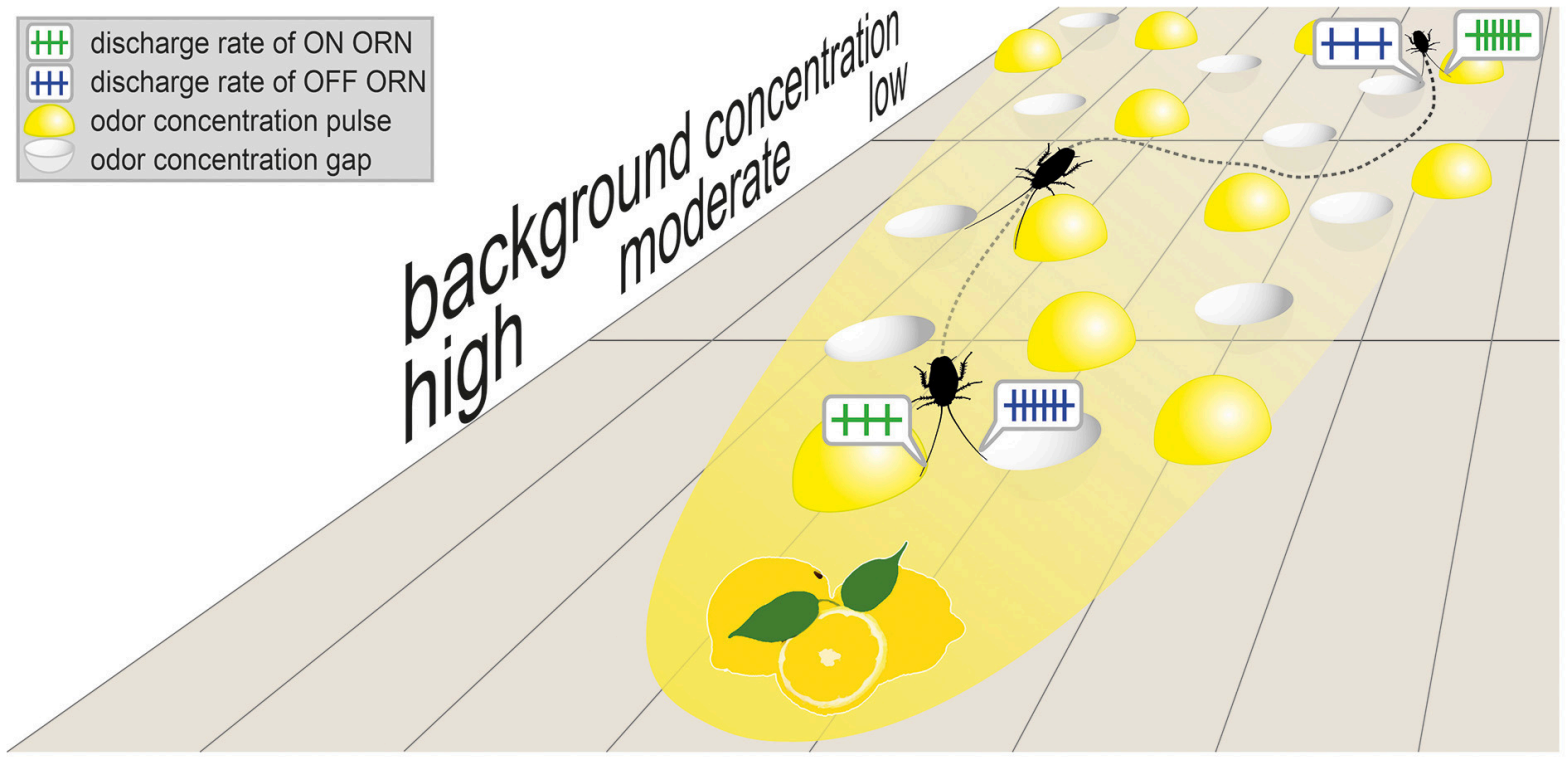
**Schematic representation of turbulent plume features proposed as potentially relevant to plume tracking, including odor pulses and odor gaps separated by the background odor concentration that gradually increases with decreasing distance to the source (lemon fruits)**. A cockroach walking toward the source will perceive the spatial distribution of odor concentration as temporal concentration change. By using simultaneous or successive sampling strategies, concentration differences determined by bilateral comparison of the responses of ON and OFF ORNs on both antennae will guide the cockroach to move forward or return to the previous position. Response bias for equal-amplitude concentration drops relative to jumps enhances detection of low concentration change with rising background level. Therefore, the OFF responses are used as alert information for accurately tracking motion.

Classical concepts of odor plume tracking use spatial and temporal sampling to explain the mechanisms underlying initiation of a response and controlling the orientation of an organism to an odor source (Vickers, 2000; Willis, 2008). Irrespectively of whether bilateral or sequential comparison of odor concentrations is used for orientation, a cockroach following a background concentration gradient should balance between the responses of the ON and OFF ORNs. Clearly, strong responses of an ON ORN indicate the direction toward the odor source. Weak responses will also do so, provided that a change in the insect's course produces a stronger response in the OFF ORN. Strong responses of the OFF ORN indicate that concentration is falling. From a perceptual perspective, falling-concentration bias results in an overestimation of drops relative to jumps. In terms of accuracy, drops are not only perceived by the cockroach as being stronger than they actually are, they also specify the location of plume edges to be closer than they are. In this view, the cockroach uses the responses of the ON ORNs for distance information and the responses of the ON ORNs as alert or warning information. From the cockroach's perspective, tendencies in concentration changes rather than exact values of concentration change suffice for responding appropriately to odor pulses and odor gaps and provide timely arrival at the odor source (Figure 4).

The discharge of ORNs to concentration jumps superimposed on different background levels have recently been described in the fruit fly. In the experiments with OR59b ORNs (Kim et al., 2011), odor stimulation consisted of a step-like sequence of 3 different concentrations of acetone. Each concentration was presented for 2 s and created the background for the next step. After an initial phasic increase to the concentration step, the ORNs displayed relatively constant rates of discharge over the 2-s stimulation period. The peak discharge to equal-amplitude acetone steps gradually decreased with increasing background level. Therefore, ORNs are unable to measure accurately the concentration change. In a study of ab3A ORNs it was shown that the peak discharge rates to 500 ms puffs of methyl butyrate, ethyl acetate and ethyl butyrate decreased with increasing background concentration (Martelli et al., 2013). However, when the discharge rates to different concentration puffs were normalized by the peak responses to the same odorant, the diminishing effect of the increasing background concentration disappeared. Moreover, the dynamics of the normalized responses did not depend on the dynamics of the brief concentration puff, even if the concentration of the odor puff was varied. This independence of the normalized dynamic responses on the dynamics of the odor puff was interpreted as being a prerequisite for ab3A ORNs to use the dynamics of their responses for mediating characteristics of the odor stimulus such as the presence of different compound in the mixture.

The inability of ORNs in insects and crustaceans to accurately measure the magnitude of concentration change is not a matter of variance of the discharge rates. It is rather the concession of their additional dependence on the background concentration. Since the ON and OFF ORNs adapt relatively slowly and only partially to the background level, the discharge rates signal relative concentration changes. ORNs adapting minimally or not at all may be capable of signaling the actual level of odor concentration. Tonic systems are well suited to convey information about unchanging concentrations, but would fail to signal concentration changes because their ORNs remain excited after the change has ceased. Such maintained discharge would distort temporal information.

## Author contributions

MH and HT conceived and designed experiments, MH performed experiments and analyzed data; MH and HT interpreted results and wrote the paper; MH prepared figures; HT edited and revised manuscript.

### Conflict of interest statement

The authors declare that the research was conducted in the absence of any commercial or financial relationships that could be construed as a potential conflict of interest.
